# Supplementary material for: Causal Relationship between Aging and Anorexia Nervosa: A White-Matter-Microstructure-Mediated Mendelian Randomization Analysis
Source: Biomedicines. 2024 Aug 16;12(8):1874. doi: 10.3390/biomedicines12081874 (PMC11351342; doi:10.3390/biomedicines12081874)
Supplement: Supplementary file 1 [file biomedicines-12-01874-s001.zip › Supplementary Figure/Figure S4/Singe SNP plot of MR analysis for TL to ubm-b-1499.pdf]

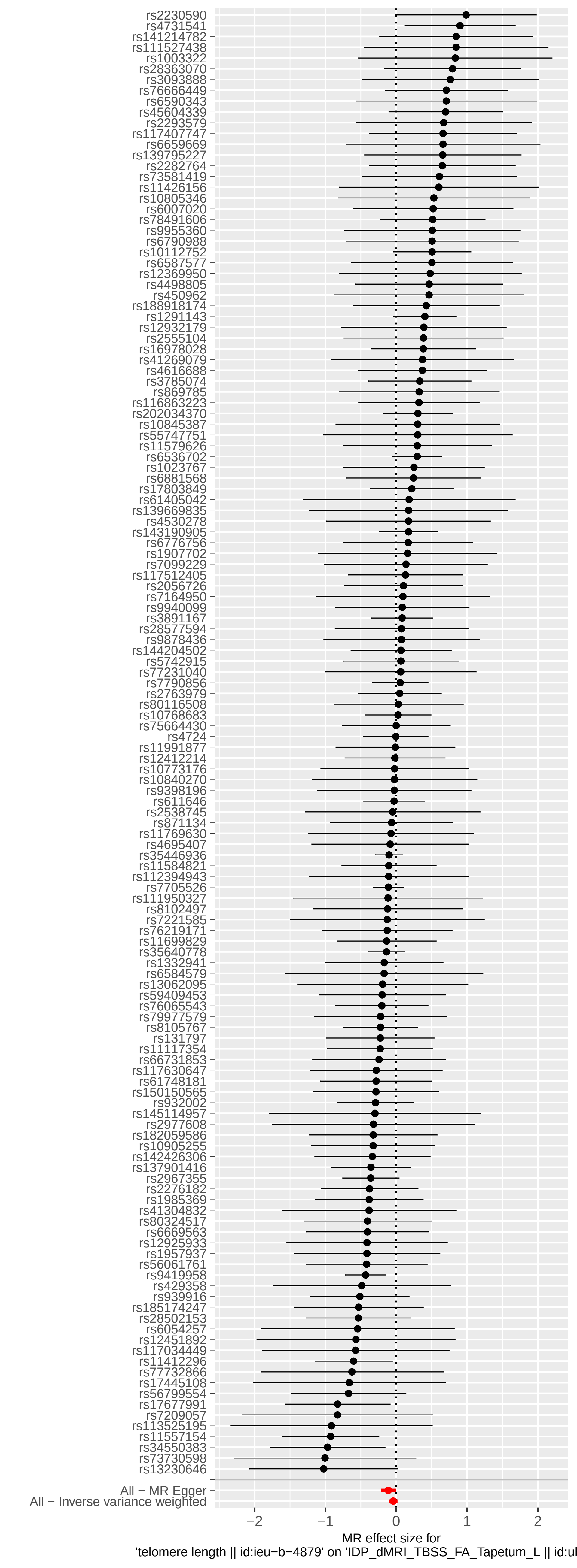
rs2230590
rs4731541
rs141214782
rs111527438
rs1003322
rs28363070
rs3093888
rs76666449
rs6590343
rs45604339
rs2293579
rs117407747
rs6659669
rs139795227
rs2282764
rs73581419
rs11426156
rs10805346
rs6007020
rs78491606
rs9955360
rs6790988
rs10112752
rs6587577
rs12369950
rs4498805
rs450962
rs188918174
rs1291143
rs12932179
rs2555104
rs16978028
rs41269079
rs4616688
rs3785074
rs869785
rs116863223
rs202034370
rs10845387
rs55747751
rs11579626
rs6536702
rs1023767
rs6881568
rs17803849
rs61405042
rs139669835
rs4530278
rs143190905
rs6776756
rs1907702
rs7099229
rs117512405
rs2056726
rs7164950
rs9940099
rs3891167
rs28577594
rs9878436
rs144204502
rs5742915
rs77231040
rs7790856
rs2763979
rs80116508
rs10768683
rs75664430
rs4724
rs11991877
rs12412214
rs10773176
rs10840270
rs9398196
rs611646
rs2538745
rs871134
rs11769630
rs4695407
rs35446936
rs11584821
rs112394943
rs7705526
rs111950327
rs8102497
rs7221585
rs76219171
rs11699829
rs35640778
rs1332941
rs6584579
rs13062095
rs59409453
rs76065543
rs79977579
rs8105767
rs131797
rs11117354
rs66731853
rs117630647
rs61748181
rs150150565
rs932002
rs145114957
rs2977608
rs182059586
rs10905255
rs142426306
rs137901416
rs2967355
rs2276182
rs1985369
rs41304832
rs80324517
rs6669563
rs12925933
rs1957937
rs56061761
rs9419958
rs429358
rs939916
rs185174247
rs28502153
rs6054257
rs12451892
rs117034449
rs11412296
rs77732866
rs17445108
rs56799554
rs17677991
rs7209057
rs113525195
rs11557154
rs34550383
rs73730598
rs13230646
All − MR Egger
All − Inverse variance weighted
−2
−1
0
1
2
MR effect size for
'telomere length || id:ieu−b−4879' on 'IDP_dMRI_TBSS_FA_Tapetum_L || id:ul
